# Different antihypertensive and metabolic responses to rostafuroxin in undernourished and normonourished male rats: Outcomes on bodily Na^+^ handling

**DOI:** 10.14814/phy2.15820

**Published:** 2023-09-04

**Authors:** Amaury Pereira‐Acácio, João P. M. Veloso‐Santos, Danilo Alves‐Bezerra, Glória Costa‐Sarmento, Humberto Muzi‐Filho, Adalberto Vieyra

**Affiliations:** ^1^ Graduate Program of Translational Biomedicine/BIOTRANS University of Grande Rio Duque de Caxias Brazil; ^2^ Carlos Chagas Filho Institute of Biophysics Federal University of Rio de Janeiro Rio de Janeiro Brazil; ^3^ National Center for Structural Biology and Bioimaging/CENABIO Federal University of Rio de Janeiro Rio de Janeiro Brazil; ^4^ National Institute of Science and Technology for Regenerative Medicine/REGENERA Rio de Janeiro Brazil

**Keywords:** arterial hypertension, chronic undernutrition, regional basic diet, renal Na^+^‐transporting ATPases, rostafuroxin, sodium and water balance

## Abstract

Hypertension is a pandemic nowadays. We aimed to investigate whether chronic undernutrition modifies the response to the antihypertensive drug rostafuroxin in juvenile hypertensive rats. Chronic undernutrition was induced in male rats using a multideficient diet known as the Regional Basic Diet (RBD), mimicking alimentary habits in impoverished regions worldwide. Animals were given RBD—or a control/CTRL normal diet for rodents—from weaning to 90 days, and rostafuroxin (1 mg/kg body mass) was orally administered from day 60 onwards. For the last 2 days, the rats were hosted in metabolic cages to measure food/energy, water, Na^+^ ingestion, and urinary volume. Rostafuroxin increased food/energy/Na^+^ intake in CTRL and RBD rats but had opposite effects on Na^+^ balance (intake minus urinary excretion). The drug normalized the decreased plasma Na^+^ concentration in RBD rats, increased urinary volume in RBD but not in CTRL, and decreased and increased urinary Na^+^ concentration in the RBD and CTRL groups, respectively. Rostafuroxin decreased the ouabain‐sensitive (Na^+^+K^+^)ATPase and increased the ouabain‐resistant Na^+^‐ATPase from proximal tubule cells in both groups and normalized the systolic blood pressure in RBD without effect in CTRL rats. We conclude that chronic undernutrition modifies the response of blood pressure and metabolic responses to rostafuroxin.

## Graphical abstract

Rostafuroxin, an antagonist of endogenous cardiotonic steroids, has different effects in rats depending on their nutritional status. In undernourished hypertensive rats, the drug exerts hypotensive, antinatriuretic, and diuretic actions. In normonourished normotensive animals, these actions are not seen; notably, the drug acts as a natriuretic agent. Rostafuroxin mobilizes sodium (Na) from non‐osmotic compartments, which are larger in undernourished hypertensive rats.

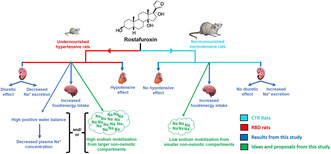

## 1 INTRODUCTION

Rostafuroxin (a digitoxigenin derivative) (Ferrari et al., 2006) is an antihypertensive agent acting as an antagonist of cardiotonic steroids, including endogenous ouabain, which main molecular target is the (Na^+^+K^+^)ATPase (Ferrandi et al., 2002). Cardiotonic steroids are central in developing cardiovascular dysfunction, including arterial hypertension (Schoner & Scheiner‐Bobis, 2007). The proposal for their existence dates back to 1885 (Ringer, 1885; Schoner, 2002) in a pioneering study of heart function.

Besides its classical active transporting action of maintaining the Na^+^ and K^+^ gradients across the plasma membrane of cells (Glynn, 2002), growing evidence in the last two decades indicates that the ouabain‐sensitive (Na^+^ +K^+^)ATPase is a central signaling transducer in a great variety of physiological and pathological conditions (for excellent reviews, see Bagrov et al., 2009; Xie & Askari, 2002). This property was why strong efforts had been developed to look for—or synthesize—compounds able to modify the interaction of the (Na^+^ +K^+^)ATPase from different organs and tissues with ouabain‐/ouabain‐like substances.

Chronic undernutrition is a pandemic growing nowadays, especially in low‐income countries and impoverished areas of developed countries (Swinburn et al., 2019), where nearly 1 billion people are undernourished or facing a severe risk of undernutrition by eating a poor‐quality diet. One widely used model of a multideficient diet for rats is that formulated based on the alimentary habits of people from several developing countries (McLaren & Pellett, 1970; Murillo et al., 1974; Pak & Araya, 1977; Ramos‐Aliaga, 1978) the so‐called Regional Basic Diet (RBD) (Teodósio et al., 1990) (Table 1) (for a recent review, see Jannuzzi et al., 2022). This pro‐hypertensive diet (Jannuzzi et al., 2022; Pereira‐Acácio et al., 2022; Silva, Monnerat‐Cahli, et al., 2014) has brown beans, manioc flour, jerked meat, and sweet potatoes as the main ingredients.

**TABLE 1:** Composition of diets.^a^

Diet	CTRL^b^	RBD^c^
Protein % (w/w)	23	8
Carbohydrate % (w/w)	41	78
Lipids % (w/w)	2.5	1.7
Na % (w/w)^d^	0.34	0.24
Fe % (w/w)	0.018	0.007
Ca % (w/w)	1.8	0.04
K % (w/w)	0.9	0.3
Energy supply kcal/100 g dry weight	278	356
Vitamin supplement	Yes	No

^a^ Reproduced from Pereira‐Acácio et al. (2022).

^b^ Control (CTRL) diet. Composition indicated by the manufacturer (Neovia Nutrição e Saúde Animal, Descalvado, Brazil).

^c^ Regional Basic Diet (RBD). Composition according to the Laboratory of Experimental and Analysis of Food (LEEAL), Nutrition Department, Federal University of Pernambuco (Teodósio et al., 1990).

^d^ Average values reported by the supplier (CTRL diet) and by Muzi‐Filho et al. (2015) (RBD). For the determinations of Na^+^ intake and Na^+^ balance in the present study, the dietary Na^+^ content was measured in each food sample by flame photometry (see Materials and Methods, Section 2.6).

Undernutrition, especially chronic undernutrition, has been associated with renal and cardiovascular pathologies, including arterial hypertension (Eroğlu, 2019; Global Burden of Diseases, Injuries and Risk Factors Study/GBD, 2019; Lu et al., 2022). These studies have shown that unhealthy diets and poor nutrition are among the main risk and aggravating factors for these diseases worldwide.

Thus, the current hypothesis of this study was that RBD‐induced chronic undernutrition in hypertensive rats could modify the response of blood pressure, food and caloric intake, fluid and Na^+^ handling, and active renal Na^+^ transport to the antihypertensive drug rostafuroxin. We hypothesized that, in RBD‐fed rats, the hyperactivity of the local renin‐angiotensin‐aldosterone system (RAAS) in renal tissue (Silva, Muzi‐Filho, et al., 2014) could underpin exacerbated changes in the metabolic, renal, and cardiovascular responses to the drug. To address this hypothesis, the aims of the study were as follows. (1) to compare the influence of rostafuroxin on the blood pressure of undernourished/hypertensive and normonourished/normotensive male rats; (2) to investigate, in the same groups, the effect of rostafuroxin on dietary, energy, fluid and Na^+^ intake, plasma Na^+^ concentration, as well as on bodily water and Na^+^ balance (intake minus excretion); and (3) to compare the influence of rostafuroxin on the activity of the Na^+^‐transporting ATPases from renal proximal tubule cells of undernourished and normonourished animals.

## 2 MATERIALS AND METHODS

### 2.1 Ethical statement

The experimental procedures were approved by the Committee for Ethics in Animal Experimentation of the Federal University of Rio de Janeiro (protocol 012/19). They were performed following the Committee's Guidelines, which follow the Uniform Requirements for Manuscripts Submitted to Biomedical Journals published by the International Committee of Medical Journal Editors. The animal study is reported following ARRIVE guidelines (Percie du Sert et al., 2020).

### 2.2 Diets

The Regional Basic Diet (RBD) is a model of a multideficient diet (Teodósio et al., 1990) that mimics the alimentary habits of vast, impoverished regions of Northeast Brazil and worldwide (McLaren & Pellett, 1970; Murillo et al., 1974; Pak & Araya, 1977; Ramos‐Aliaga, 1978; Teodósio et al., 1990); its composition is given in Table 1. RBD was prepared under bromatological control in a homemade process using the facilities of the Laboratory of Food Analysis and Processing of the Josué de Castro Institute of Nutrition at the Federal University of Rio de Janeiro. The ingredients were cooked, oven‐dried at 60°C, and ground before mixing. Water was added to form a sticky wet mass cut in small pieces—as the standard commercial control diet (CTRL)—and finally dehydrated for 1 day at 60°C. The composition of the CTRL diet (Neovia Nutrição e Saúde Animal, Descalvado, Brazil) (Table 1) follows the recommendations of the American Institute of Nutrition for rodents (AIN‐93G) (Reeves, 1997). The dietary Na+ content was determined by flame photometry after acid extraction with 1 N HNO_3_. The minerals (K^+^, Ca^2+^, and Fe^2+^) and vitamin content were those encountered after mixing the dietary components, as previously determined (Perkin‐Elmer Corporation, 1968; Sandel, 1959; Snell & Snell, 1967; Teodósio et al., 1990).

### 2.3 Experimental groups

Female Wistar rats (*n* = 20) were kept and mated (4 females:1 male) in the Vivarium of Neglected Diseases and Undernutrition of the Carlos Chagas Filho Institute of Biophysics, Federal University of Rio de Janeiro. Male offspring were weaned at 28 days of age. Since animal and human studies have shown differences in the mechanisms responsible for blood pressure control between the sexes, we only used male rats (Bedran‐de‐Castro & Bedran‐de‐Castro, 1992; Reckelhoff, 2018). The animals were randomly divided into two groups: one received the RBD ad libitum, and the other received the CTRL diet until they reached 90 days of age. Drinking water was also offered ad libitum. When the rats reached 60 days, the two groups were randomly subdivided into two other subgroups, originating with those that received Rostafuroxin (Rosta: 1 mg/kg body mass diluted in 99% ethanol; Aobious Inc.) or the same volume of water. Thus, the four experimental groups were: CTRL, CTRL+Rosta, RBD, and RBD + Rosta. Six rat litters were analyzed during the study. In the first one, the influence of the vehicle was investigated from day 60 (25 μL in CTRL and 10 μL in the RBD group, daily). Since no effect was found on body mass and final values of blood pressure, the vehicle was not administered to untreated CTRL and RDB rats.

A 24 h‐acclimation period in the metabolic cages occurred from day 88 (morning) to day 89 (morning), and a 24 h‐recording period of water ingestion, food ingestion, and urinary volume started on this day. After this period, the blood pressure was measured. Finally, the rats were euthanized by decapitation, the blood was collected under EDTA, and the plasma was immediately separated to measure the plasma Na^+^ concentration ([Na^+^]_pls_). The kidneys were carefully dissected to obtain plasma membrane preparations from proximal tubule cells.

### 2.4 Measurement of blood pressure

Systolic blood pressure was measured by tail‐cuff plethysmography (Feng et al., 2008) in conscious rats immediately after the cage period (day 90) by using the Insight system model V2.01 (Bonther), coupled with the appropriate software (Pressure Gauge 2.0, Insight). Acclimation in a heated chamber (32°C) for 15 min preceded the blood pressure recordings, and the recordings were only taken when the rats did not present sudden movements. Five determinations were made for each rat, and the average of the five values was used.

### 2.5 Preparation of plasma membrane‐enriched fraction from kidney proximal tubule cells

After the removal of the kidneys, the membrane preparations were obtained by homogenization and differential centrifugations of tissue fragments from the outer region of the renal cortex (*cortex corticis*) (Whittembury & Proverbio, 1970), where the cell population corresponds to >95% of proximal tubules. The preparation procedure was as described by Silva, Monnerat‐Cahli, et al., 2014. Protein content was determined using the method described by Lowry et al. (1951). The samples were used to measure the activity of the two Na^+^‐transporting ATPases, the ouabain‐sensitive (Na^+^+K^+^)ATPase, and the ouabain‐resistant, furosemide‐sensitive Na^+^‐ATPase (Silva, Monnerat‐Cahli, et al., 2014).

### 2.6 Determination of Na^+^ in food, plasma, and urine samples

Na^+^ content (food) and Na^+^ concentrations (plasma and urine; [Na^+^]_pls_ and [Na^+^]_ur_) were determined by flame photometry (Analyzer, São Paulo, Brazil) using a standard solution containing 140 mequiv Na^+^/L (Analyzer). The Na^+^ content in the diets was determined after acid extraction (5 mL 1 N HNO_3_:0.5 g powdered food). The suspension was stirred for 48 h at room temperature and allowed to sediment for 24 h to determine the Na^+^ concentration in the supernatant. The urinary Na^+^ excretion in 24 h was calculated using the urinary volume in this period and urinary Na^+^ concentration. The Na^+^ balance was calculated as the difference between Na^+^ intake and Na^+^ excretion in the same 24‐period. The Na^+^ intake was calculated from the ingestion of food and the dietary Na^+^ content.

### 2.7 Determinations of K^+^ (plasma and urine) and urea (urine)

K^+^ concentrations (plasma and urine; [K^+^]_pls_ and [K^+^]_ur_) were determined by flame photometry (Analyzer) using a standard solution containing 5 mequiv K^+^/L. The urinary concentrations of urea were measured using a commercial kit (Bioclin).

### 2.8 Estimation of urinary osmolality

We estimated the urinary osmolality from the contribution of the solutes Na^+^, K^+^, and urea, using the expression 2 × ([Na^+^]_ur_ + [K^+^]_ur_ + [urea]_ur_ (Borrego Utiel et al., 2020) because both CTRL and RBD do not present glucosuria. Since Cl^−^ is the more abundant anion in the urine, the number 2 in the expression means that the sum of [Na^+^] and [K^+^] can be assumed to be equal to [Cl^−^].

### 2.9 Determination of albumin in plasma samples

The plasma albumin concentration was measured using a commercial kit (Bioclin).

### 2.10 Determination of the activities of renal Na^+^‐transporting ATPases

The ouabain‐sensitive (Na^+^+K^+^)ATPase and the ouabain‐resistant, furosemide‐sensitive Na^+^‐ATPase activities were determined by quantifying the inorganic phosphate (P_i_) released during ATP hydrolysis (Taussky & Shorr, 1953). The activities were determined in triplicate precisely as described by Silva, Monnerat‐Cahli, et al. (2014). The activities were calculated by the differences between the values obtained in the absence and presence of 2 mM ouabain (for (Na^+^+K^+^)ATPase); and between the values obtained in the absence and presence of 2 mM furosemide, always in the presence of 2 mM ouabain (for Na^+^‐ATPase).

### 2.11 Statistical analysis

The sample size was determined in pilot studies using normonourished undernourished rats that showed intraassay and interassay coefficients of variation lower than 5%–6% for the different determinations. Blinding of body mass and systolic blood pressure measurements were not possibly due to the visible differences in body size between the CTRL and the RBD rats. Biochemical measurements were blinded because one coworker prepared the samples and other(s) carried out the determinations. Randomizations of rats from the different litters were based on single sequences of random assignments. Randomizations of rats for rostafuroxin treatment did not occur in the separated CTRL and RBD‐fed groups. Normal distribution was checked using the Shapiro–Wilks test. We found some data that followed a normal distribution, and others did not. We assessed differences using two‐way ANOVA with Bonferroni's posttest when the data follow normal distribution and *n* > 4, and the Kruskal–Wallis test followed by Dunn's test for selected pairs when the data do not follow normal distribution, as indicated in the figure legends. Statistical analyses were carried out using GraphPad Prism 8 software (version 8.02, GraphPad Software, Inc.). Results are expressed as mean ± SD. Significant differences were set at *p* < 0.05.

## 3 RESULTS

### 3.1 Body mass, food, energy, Na^+^, and water intake in undernourished rats: Effects of rostafuroxin administration

**FIGURE 1:**
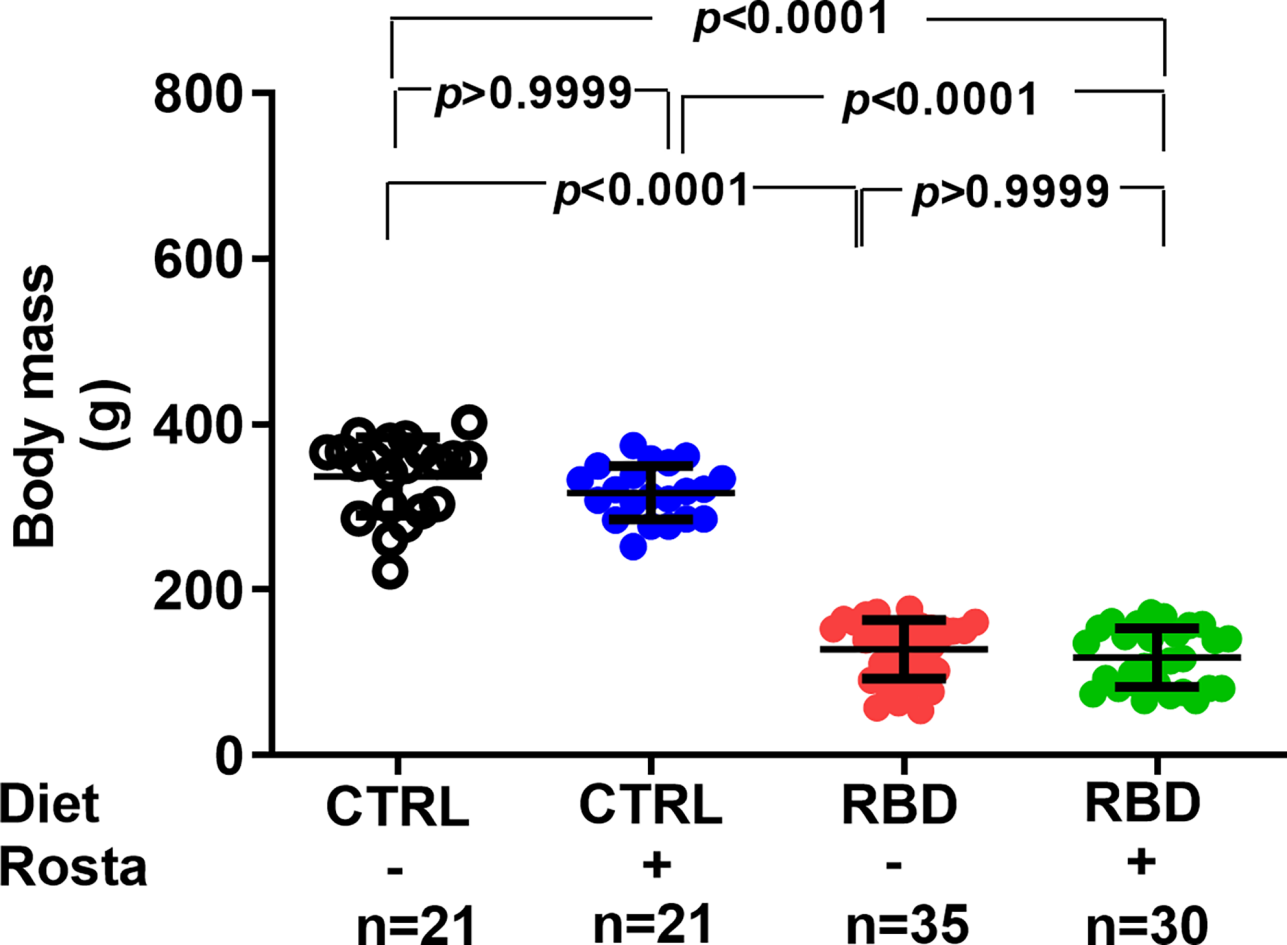
Body mass of Control (CTRL) and Regional Basic Diet (RBD) rats at 90 days of age. Diets and treatment with rostafuroxin (Rosta) or not are indicated on the *abscissa*. The dispersed ensemble of points for each experimental group is accompanied by horizontal lines that indicate mean ± SD. The data did not follow normal distribution according to the Shapiro–Wilks test. Differences were assessed using the Kruskal–Wallis test followed by Dunn's test for selected pairs. The number of animals is given on the *abscissa* beneath the corresponding column of points; *p* values are indicated within the figure.

**FIGURE 2:**
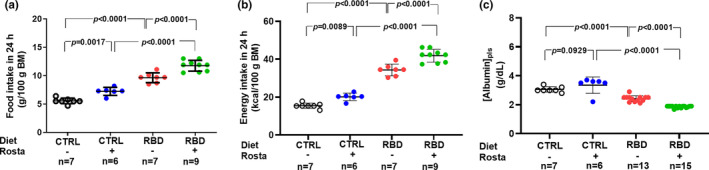
Food intake (a), energy intake (b), and plasma albumin concentration (c) measured between 89 and 90 days of age. Diets and treatment with rostafuroxin or not are indicated on the *abscissae*. Abbreviations are as in the legend in Figure 1. The dispersed ensembles of points for each variable and experimental group are accompanied by horizontal lines that indicate mean ± SD. All the data follow normal distribution according to the Shapiro–Wilks test. Differences were assessed using two‐way ANOVA followed by Bonferroni's test. The number of animals is given on the *abscissae* beneath the corresponding column of points; *p* values are indicated within the panels.

Figure 1 shows the accentuated diminution of body mass (~60%) in the group that received RBD from weaning at 28 days until 90 days of age compared with CTRL. The administration of rostafuroxin during the previous 30 days was without effect on the body mass of both groups. When food intake was evaluated at the same age (Figure 2a), we observed an increase (approximately 70%) in RBD rats with respect to CTRL and that rostafuroxin administration provoked an augment in both groups. The combination of higher food intake with the higher energy content of the deficient diet (Table 1) resulted in approximately 110% higher energy intake by RBD rats, and, also as expected from the food intake data, the rostafuroxin‐treated rats incorporated much dietary energy in both CTRL and RBD rats (Figure 2b). The undernutrition of RBD rats is reflected in their decreased plasma albumin concentration ([albumin]_pls_) (Figure 2c) in comparison to the CTRL level. Rostafuroxin administration decreased [albumin]_pls_ in the RBD group, without influence in CTRL rats.

**FIGURE 3:**
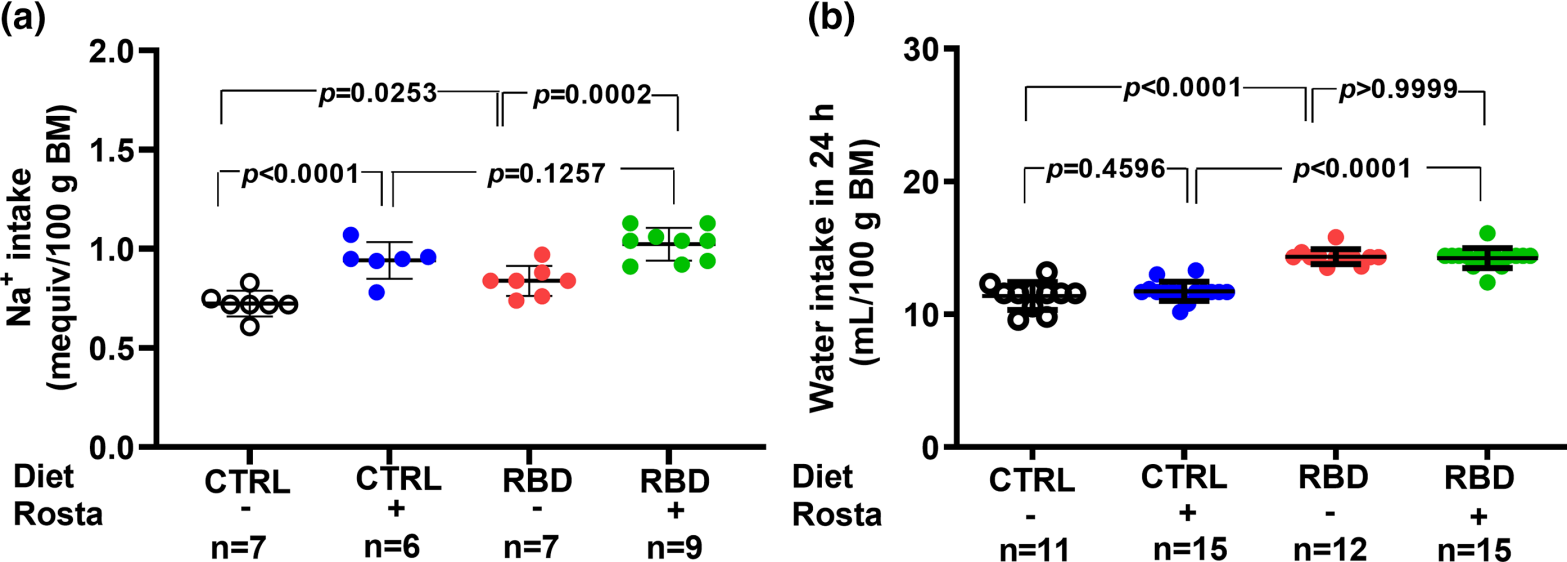
Na^+^ intake (a) and water intake (b) between days 89 and 90. The Na^+^ intake in 24 h was calculated from the dietary Na^+^ content and the food intake. Diets and treatment with rostafuroxin or not are indicated on the *abscissae*. Abbreviations are as in the legend in Figure 1. The dispersed ensembles of points for each variable and experimental group are accompanied by horizontal lines that indicate mean ± SD. All the data follow normal distribution according to the Shapiro–Wilks test. Differences were assessed using two‐way ANOVA followed by Bonferroni's test. The number of animals is given on the *abscissae* beneath the corresponding column of points; *p* values are indicated within the panels.

Despite the lower Na^+^ content of the RBD, the Na^+^ intake was higher in the undernourished rats compared with the CTRL (*p* = 0.0253), and the administration of rostafuroxin resulted in a significant augment of salt ingestion in the two groups (30% in CTRL and 20% in RDB; Figure 3a). The analysis of water ingestion per 100 g body mass (Figure 3b) reveals that RBD rats drank much water in the same period and that rostafuroxin had no effect in either group.

### 3.2 Urinary Na^+^ excretion, urinary volume, urinary osmolality, Na^+^ balance, water balance, and plasma Na^+^ concentration in undernourished rats: Effects of rostafuroxin

**FIGURE 4:**
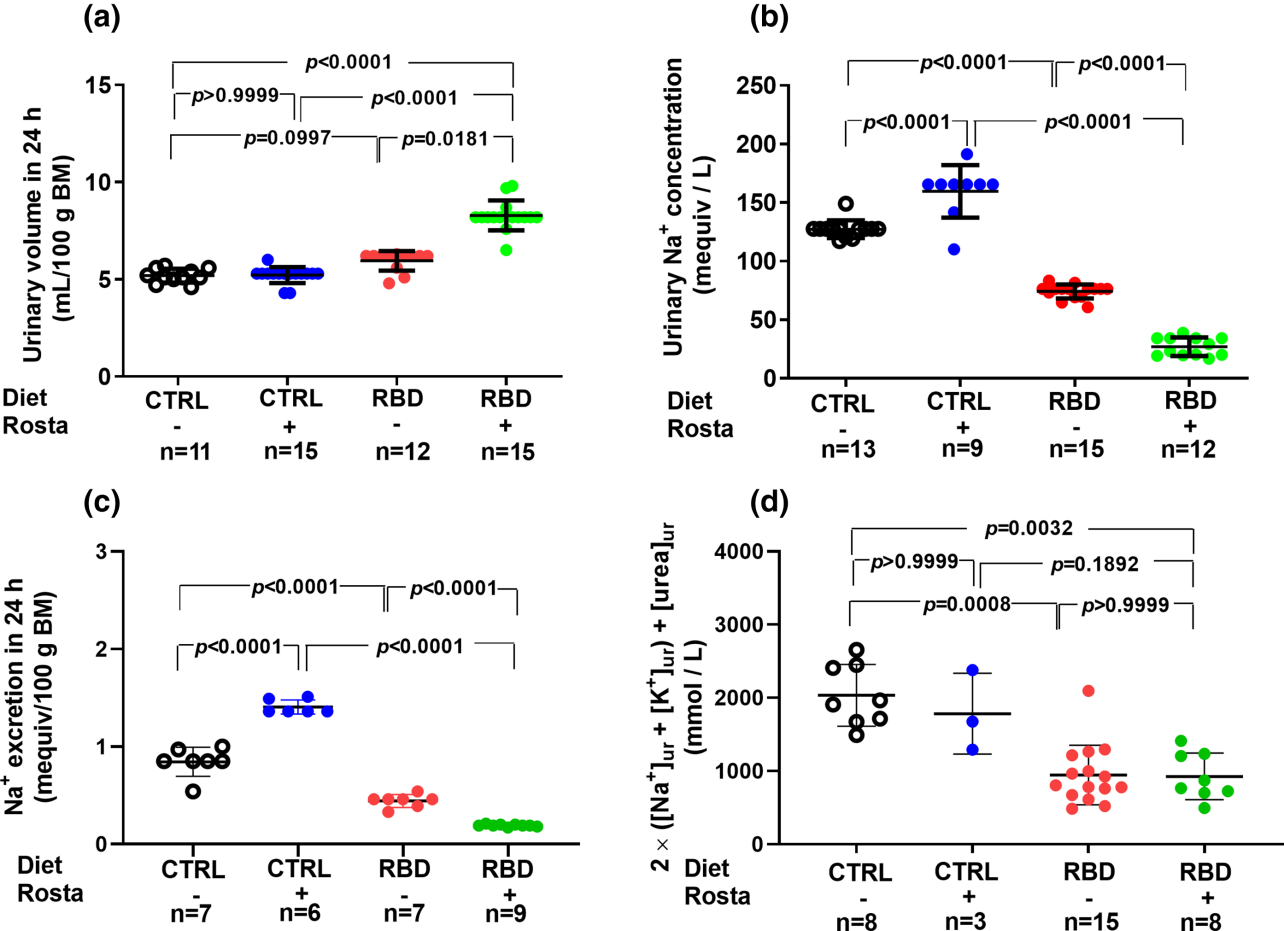
Urinary volume (a), urinary Na^+^ concentration (b), Na^+^ excretion in 24 h (c), and estimated urinary osmolality (d) measured between days 89 and 90. Diets and treatment with rostafuroxin or not are indicated on the *abscissae*. Abbreviations are as in the legend in Figure 1. The dispersed ensembles of points for each variable and experimental group are accompanied by horizontal lines that indicate mean ± SD. The data did not follow normal distribution according to the Shapiro–Wilks test. Differences were assessed using the Kruskal–Wallis test followed by Dunn's test for selected pairs. The number of animals is given on the *abscissae*; *p* values are indicated within the panels.

The urinary Na^+^ excretion per 100 g body mass in 24 h of rats aged 90 days was calculated from the urinary volume in the same period and the urine Na^+^ concentration ([Na^+^]_ur_). The urinary volume per 100 g body mass (Figure 4a) was slightly but significantly higher in RBD than in CTRL rats, and rostafuroxin provoked extra diuresis in the undernourished animals without influence in the CTRL group. The analysis of [Na^+^]_ur_ (Figure 4b) revealed a 40% lower value in the RBD rats compared with CTRL and the opposite effects of rostafuroxin depending on the nutritional status: while the drug promoted a 25% increase in [Na^+^]_ur_ in normonourished rats, it provoked a diminution of near 50% in the undernourished group. The urinary Na^+^ excretion in 24 h (Figure 4c) was 50% lower in RBD rats compared with CTRL rats. Furthermore, the influence of rostafuroxin reflects that found with [Na^+^]_ur_: a significant increase in normonourished rats and a pronounced decrease in the undernourished group. Figure 4d shows that urinary osmolality is lower in RBD rats than in CTRL rats, without the effect of rostafuroxin in both groups.

**FIGURE 5:**
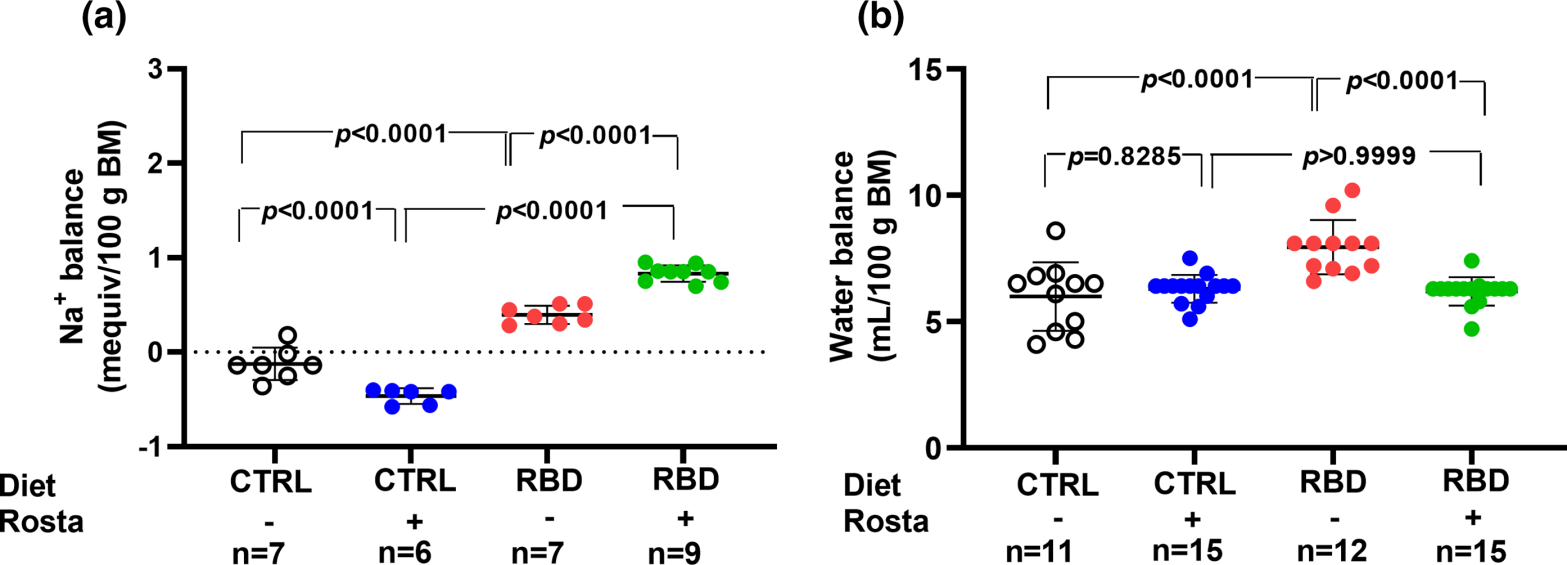
Na^+^ balance (a) and water balance (b). The Na^+^ balance was calculated as the difference between Na^+^ intake and urinary Na^+^ excretion in 24 h between days 89 and 90. The water balance was calculated as the difference between water intake and urinary volume in 24 h recorded between days 89 and 90. Diets and treatment with rostafuroxin or not are indicated on the *abscissae*. Abbreviations are as in the legend in Figure 1. The dispersed ensembles of points for each variable and experimental group are accompanied by horizontal lines that indicate mean ± SD. All the data in (a) and (b) follow normal distribution according to the Shapiro–Wilks test. Differences were assessed using two‐way ANOVA followed by Bonferroni's test. The number of animals is given on the abscissae; *p* values are indicated within the panels.

**FIGURE 6:**
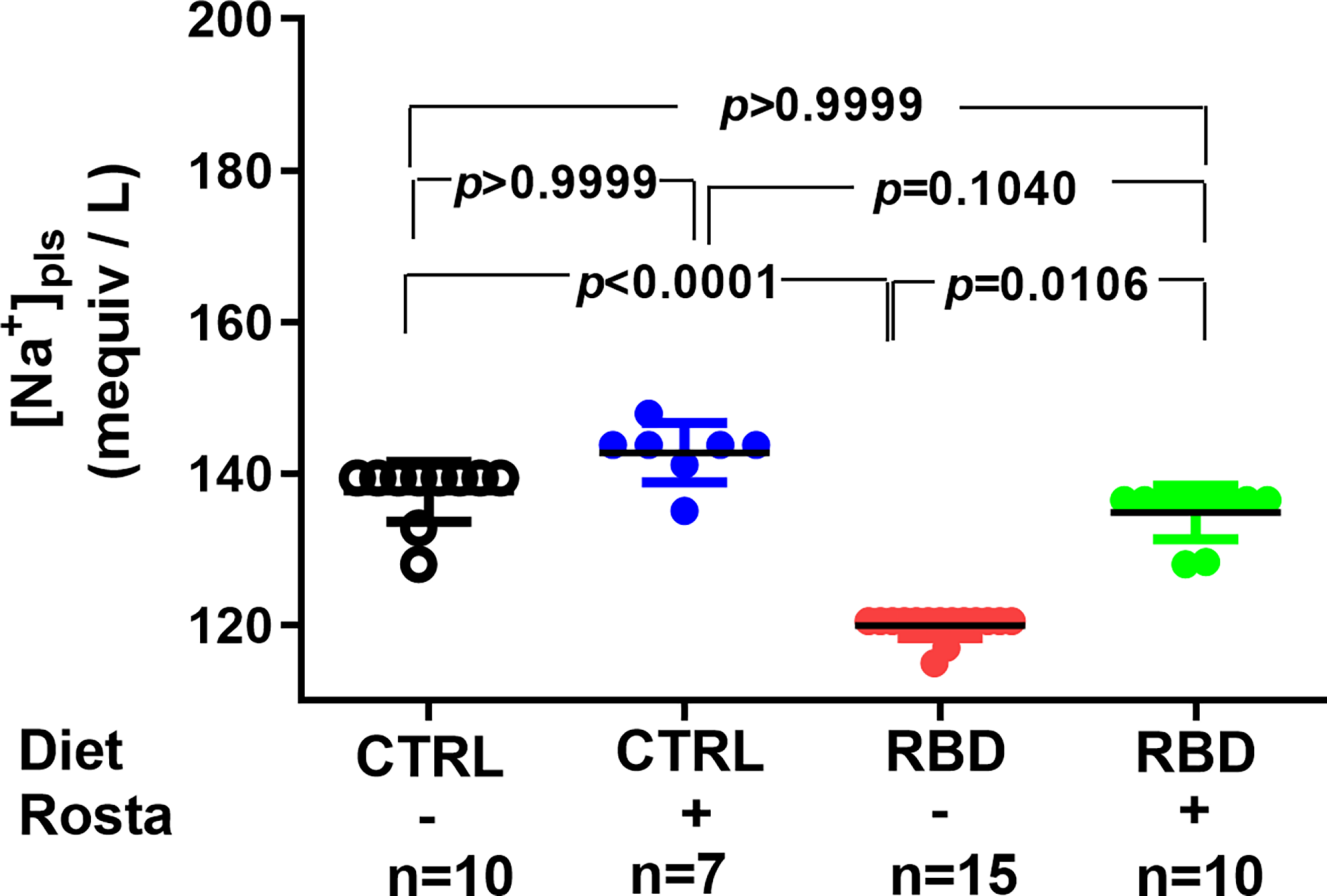
Plasma Na^+^ concentration ([Na^+^]_pls_). Measurements were carried out in samples collected on day 90. Diets and treatment or not with rostafuroxin are indicated on the *abscissa*. Abbreviations are as in the legend in Figure 1. The dispersed ensemble of points for each experimental group is accompanied by horizontal lines that indicate mean ± SD. The data did not follow normal distribution according to the Shapiro–Wilks test. Differences were assessed using the Kruskal–Wallis test followed by Dunn's test for selected pairs. The number of animals is given on the abscissa; *p* values are indicated within the panels.

The Na^+^ balance at the same age, calculated as daily Na^+^ ingestion minus urinary Na^+^ excretion in the same 24 h‐period, is seen in Figure 5a. When housed in cages, the CTRL rats presented with a slightly negative Na^+^ balance of approximately −0.10 mequiv/100 g in 24 h, which was more negative (−0.50 mequiv/100 g in 24 h) when these animals were given rostafuroxin. In contrast, Na^+^ balance was positive in the RBD, and the administration of rostafuroxin provoked a 100% increase. The water balance was calculated as the difference between water intake and urinary volume, and it is presented in Figure 5b. Except for the case of RBD rats (8 mL in 24 h), all animals had a daily positive water balance of 6 mL. Concerning plasma Na^+^ concentration ([Na^+^]_pls_), Figure 6 shows that it decreased to an average value of 120 mequiv/L in RBD rats from 139 mequiv/L encountered in the CTRL group. Rostafuroxin did not modify [Na^+^]_pls_ in normonourished rats but promoted an elevation in RBD rats, reaching an average value that matched that of CTRL.

### 3.3 Na^+^‐transporting ATPases from proximal tubule cells and systolic blood pressure: Effects of rostafuroxin

**FIGURE 7:**
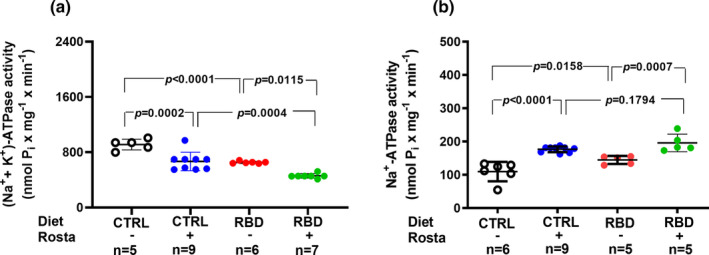
Renal proximal tubules Na^+^‐transporting ATPases. Determinations were carried out in plasma membrane‐enriched preparations isolated from the outermost region of the renal cortex (*cortex corticis*) at day 90. Diets and treatment or not with rostafuroxin are indicated on the *abscissae*. Abbreviations are as in the legend in Figure 1. (a) Ouabain‐sensitive (Na^+^+K^+^)ATPase. (b) Ouabain‐resistant Na^+^‐ATPase. The dispersed ensemble of points for each ATPase and experimental group is accompanied by horizontal lines that indicate mean ± SD. The data in (a) and (b) follow normal distribution according to the Shapiro–Wilks test. Differences were assessed using two‐way ANOVA followed by Bonferroni's test. The number of animals is given on the *abscissae*; *p* values are indicated within the panels.

The active Na^+^ transport across the proximal renal tubules is a crucial process in regulating Na^+^ content and fluid balance in the different organs and liquid compartments (McDonough, 2010; Zhuo & Li, 2013). Figure 7 shows the opposite influence of the nutritional status and rostafuroxin administration on the two Na^+^‐transporting ATPases present in the basolateral membranes of proximal tubule cells (Silva, Monnerat‐Cahli, et al., 2014). The activity of the main pump, the ouabain‐sensitive (Na^+^+K^+^)ATPase (Figure 7a), decreased by 30% in RBD rats in comparison to CTRL, as previously described (Pereira‐Acácio et al., 2022), with rostafuroxin promoting a further decrease in both groups. In the case of the ouabain‐resistant, furosemide‐sensitive Na^+^‐ATPase (Figure 7b), undernutrition provoked a 50% increment in its activity, equalized at the same higher levels in CTRL+Rosta and RBD + Rosta after further upregulation in the animals given the drug.

**FIGURE 8:**
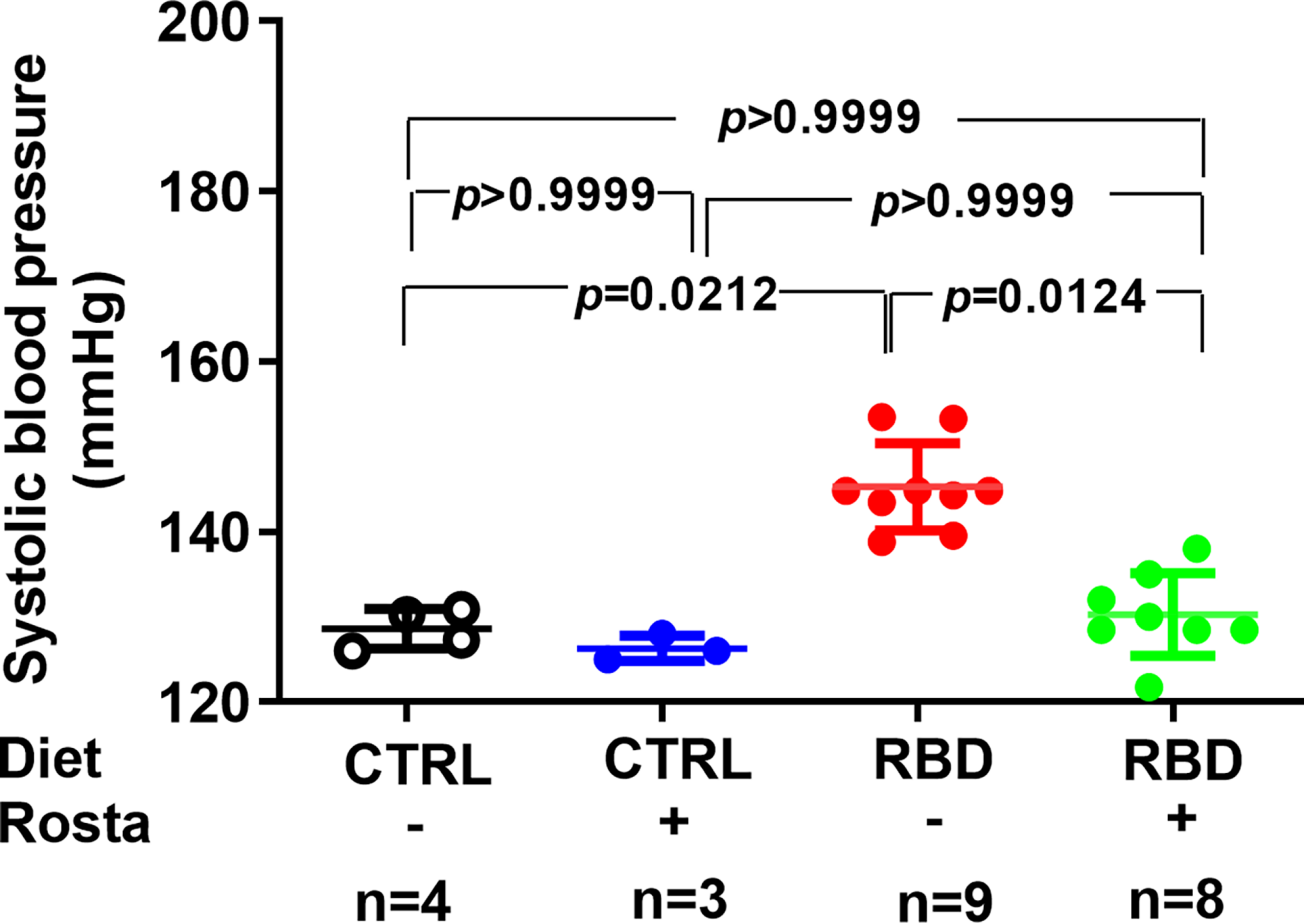
Systolic blood pressure. Systolic blood pressure was measured at day 90. Diets and treatment or not with rostafuroxin are indicated on the *abscissa*. Abbreviations are as in the legend in Figure 1. The dispersed ensembles of points for each experimental group are accompanied by horizontal lines that indicate mean ± SD. The data did not follow normal distribution according to the Shapiro–Wilks test. Differences were assessed using the Kruskal‐Wallis test followed by Dunn's test for selected pairs. The number of animals is given on the *abscissae*; *p* values are indicated within the panels.

Figure 8 shows that systolic blood pressure was elevated in RBD rats, which was normalized by the administration of rostafuroxin. In contrast, the drug did not modify the pressoric levels of the CTRL rats. RBD‐fed rats were hypertensive at days 55–60, as described elsewhere (Mendes et al., 2017). The time course of hypertension in these animals had three periods: (1) pre‐hypertensive (raising blood pressure from 44 to 52 days without differences with CTRL rats; (2) onset of hypertension (from 52 to 63 days, the period in which the systolic blood pressure increases and becomes statistically different from that recorded in CTRL rats); (3) established hypertension (from day 63 onwards) (Mendes et al., 2017). Due to this time course, we started rostafuroxin administration on day 60.

Since RBD has low K^+^ content (Table 1) and hypokalemia induces general vasoconstriction and hypertension (Haddy et al., 2006; Suga et al., 2001), we measured plasma K^+^ concentration in the four groups. We obtained the following values (means ± SEM in mequiv/L): 7.0 ± 0.2 (CTRL, *n* = 7), 6.2 ± 0.3 (CTRL + Rosta, *n* = 4), 6.2 ± 0.2 (RBD, *n* = 13), 6.8 ± 6.3 (RBD + Rosta, *n* = 12). Since the data distribution was nonnormal (Shapiro–Wilk's test), the means were compared using Kruskal‐Wallis test and Dunn's post‐hoc test. No differences were found among the different groups.

## 4 DISCUSSION

The central findings of the present study are that chronic administration of rostafuroxin—which was initially described as an antihypertensive drug with an inhibitory effect on the (Na^+^+K^+^)ATPase from the kidney medulla of hypertensive rats (Ferrandi et al., 2002)—modulates blood pressure, food/energy intake, protein metabolism, body Na^+^ handling, fluid balance, and the Na^+^‐transporting ATPases resident in renal proximal tubule cells of male rats, in a way that depends on the nutritional status. Even though the caloric intake in RBD rats is higher than in CTRL animals, they are considered undernourished because they are stunting (low height for age) and underweight (low weight for age) and have micronutrients insufficiencies (World Health Organization, 2021). In rats, height is the length from the tip of the nose to the base of tail (Sobel, 1978).

We induced chronic undernutrition from weaning until a rat juvenile age (Agoston, 2017) by offering ad libitum a multideficient diet, RBD, which mimics those widely used in countries and regions where undernutrition is endemic (Jannuzzi et al., 2022; McLaren & Pellett, 1970; Murillo et al., 1974; Pak & Araya, 1977; Ramos‐Aliaga, 1978; Teodósio et al., 1990). The content of proteins is very low (Table 1), and their quality is poor because their sources are beans and jerked beef, and vitamins supplementation also does not exist. These are the reasons by which the rats body mass has markedly decreased (Figure 1). In these undernourished animals compensatory mechanisms, possibly involving neuronal circuits in the central nervous system (Farr et al., 2016; Morton et al., 2006; Schwartz et al., 2000), culminated with increased food and energy intake (Figure 2a,b). This neuronal network appears to be activated by rostafuroxin, and the stimulatory effect could involve steps in which the (Na^+^+K^+^)ATPase participates (Kurita et al., 2015; Otero‐Rodiño et al., 2019) being modulated by endogenous ouabain in association with tissular Na^+^ levels (Pavlovic, 2020; Schoner & Scheiner‐Bobis, 2007).

Somewhat different results regarding the potential effects of endogenous ouabain on body mass, food intake, and Na^+^ intake were reported more than two decades ago (Lewis et al., 2014; Simonini et al., 2018; Tordoff, 1996). The chronic infusion of ouabain in rats increased body mass and had no effect on food or Na^+^ ingestion (Tordoff, 1996); however, these observations can be explained because ouabain of external origin and endogenous ouabain seems not to be the same compound or, in other words, that endogenous digitalis did not represent authentic ouabain (Lewis et al., 2014; Simonini et al., 2018). Additionally, rostafuroxin could activate other structures beyond the central nervous system, for example, the hypothalamic–pituitary–adrenal axis, which stimulates the secretion of glucocorticoids that augment appetite and food intake (Chao et al., 2017; Torres & Nowson, 2007). Other novel metabolic effects of rostafuroxin reported in the present study are those encountered in [albumin]_pls_: decrease in RBD rats without influence in the CTRL group (Figure 2c). It could be that the drug's effect on liver protein metabolism depends on the upregulation of the AMP kinase‐mediated cellular energy metabolism sensing in the liver, an organ in which AMP kinase plays a central regulatory role (Foretz & Viollet, 2018; Viollet et al., 2006).

At this point, it is relevant to note that rostafuroxin‐treated CTRL and RBD rats consume a significantly higher amount of Na^+^ (Figure 3a) and, since there was an accentuated lower [Na^+^]_pls_ in the undernourished group (minus ~20 mequiv/L) (Figure 6), two mechanisms could be responsible for these observations. First, it could be that—in the case of RBD rats—water accumulation (Figure 5b) and plasma dilution account for the decreased [Na^+^]_pls,_ an idea that is supported by the expanded plasma compartment of these rats (Silva, Monnerat‐Cahli, et al., 2014). Alternatively, it could be speculated that higher Na^+^ is present—in a greater amount in RBD rats—in silent nonosmotic tissular compartments, which was hypothesized some years ago (Titze et al., 2002, 2003) and recently revisited (Canaud et al., 2019). The increase in [Na^+^]_pls_ found in rats given rostafuroxin is compatible with the mobilization of Na^+^ from these deposits by the drug in a more accentuated way in RBD rats.

The antihypertensive effect of rostafuroxin was described as not associated with diuretic effects, despite its accentuated natriuretic action (Ferrandi et al., 2002; Nesher et al., 2009) and does not have the usual side effects of diuretics in humans, as demonstrated in clinical trials (Citterio et al., 2021). However, in the case of undernourished rats, we found a clear rostafuroxin‐induced diuretic effect (Figure 4a), possibly as the result of an altered medullary action of the drug (Ferrandi et al., 2002) in this group of rats. This observation allows us to conclude that, again, the nutritional status of the animals influences one important effect of endogenous ouabain. Since the pioneering studies mentioned above were carried out with normonourished rats (Ferrandi et al., 2002; Nesher et al., 2009), the lack of diuretic action may only occur in properly nourished animals. Facing these findings, we investigated whether rostafuroxin modified water balance in rats as the result of induced modifications in water intake and urinary volume and if—as in the case of food/energy intake—there was an influence on the nutrition status. The increased ingestion of water by RBD rats compared with CTRL animals (Figure 3b) possibly represents a regulatory response facing the high ingestion of food (solutes) (Figure 2a). In contrast with that found in the case of food/energy intake, however, no rostafuroxin‐induced polydipsia was found in both groups of animals, a result suggesting that hypothalamic circuits at the forebrain (Leib et al., 2016) which control thirst, have not (Na^+^+K^+^)ATPase as central signaling machinery, even though the intravenous injection of ouabain decreases water intake in rats (Bergmann et al., 1967). It also may be that abnormally high endogenous cardiotonic steroid levels during undernutrition affect the central responses to antidiuretic hormone and norepinephrine, as well as those to angiotensin II, at the level of neural pathways controlling thirst and hunger (Forrester et al., 2018; McKinley et al., 2019; Tan et al., 2022; Yosten & Samson, 2014).

Rostafuroxin‐induced natriuresis was one of the more noticeable effects described early (Ferrandi et al., 2002; Nesher et al., 2009). This action was attributed to a counteracting influence on circulating endogenous ouabain on renal (Na^+^+K^+^)ATPase, principally that localized in the external medulla (Ferrandi et al., 2002), because of the role of the (Na^+^+K^+^)ATPase resident in the basolateral membranes of the thick ascending segment of Henle loop (Jørgensen et al., 1971) in the final control of Na^+^ reabsorption and salt excretion at different osmolal concentrations in the tubular fluid and in the interstitium (Rocha & Kokko, 1973). This picture was confirmed in the case of CTRL rats, which presented with increased [Na^+^]_ur_ and Na^+^ excretion in 24 h when submitted to a chronic administration of rostafuroxin. However, a contrasting effect was encountered in RBD rats: their diminished [Na^+^]_ur_ and Na^+^ excretion in 24 h was further decreased by rostafuroxin (Figure 4b,c). As proposed above, the undernourished rats may present with augmented endogenous ouabain systemic production, including that in the central nervous system, that could cause increased nerve renal activity, thus eliciting an antinatriuretic response (Lim et al., 2000). In this condition, abnormal activation of the renal nerve could be the underlying basis of the opposite, strong antinatriuretic influence of rostafuroxin, which actions are dose‐dependent and also dependent on the ouabain levels when this drug is infused (Ferrandi et al., 2004). Interestingly, urinary osmolality is lower in RBD rats and this parameter was not affected by rostafuroxin (Figure 4d), suggesting that its final adjustment in the medullary portion of the nephron is not modulated by cardiotonic steroids. Moreover, when the total daily osmolytes excretion is calculated using the urinary volume, and then corrected by the corresponding average body mass of the four groups, the average resulting values 3139, 2966, 4429, and 6511 for the groups CTRL, CTRL + Rosta, RBD, and RBD + Rosta, respectively, match well with the profile of daily food intake (Figure 4a).

The results regarding [Na^+^]_ur_ and Na^+^ excretion in 24 h presented in Figure 4b,c reverberate in the bodily Na^+^ balance depicted in Figure 5a, where, in which, as expected, the influence of nutritional status clearly appears. The untreated CTRL rats had an overall Na^+^ balance that approached zero, as usually occurs with part of the rat population hosted in metabolic cages for a short period (Brensilver et al., 1985). When rostafuroxin was given to these animals, a significantly negative daily balance (~0.6 mequiv Na^+^ per 100 g BM in 24 h) was found, despite the increased Na^+^ intake (Figure 3a). This trend also suggests that rostafuroxin mobilizes the ion from a non‐osmotically active compartment (Titze et al., 2014), transiently passing through a plasma compartment slightly concentrated with Na^+^ when compared with untreated RBD rats, as seen in Figure 6. Following this line of thought, the daily positive balance means a cumulative Na^+^ storage in RBD rats along the experimental period of the assay (62 days). This positive balance doubled in the rats that received rostafuroxin, likely due to an abnormal renal response to the drug in a condition of probably elevated endogenous ouabain, mimicking the experimental antinatriuretic conditions established by Lim et al. (2000).

In a recent study (Pereira‐Acácio et al., 2022) and here (Figure 7a,b), we demonstrated that chronic undernutrition provoked by the continued administration of RBD to male rats downregulates the ouabain‐sensitive (Na^+^+K^+^)ATPase and upregulates the ouabain‐resistant Na^+^‐ATPase, the two Na^+^‐transporting ATPases that are responsible for the proximal renal reabsorption of more than 70% of the Na^+^ filtered in the glomeruli (Eaton & Pooler, 2018; Vieyra et al., 2016). Even though the hydrolytic activity of (Na^+^+K^+^)ATPase in membrane preparations does not quantitatively reflect the transport stoichiometry in intact cells, it is possible to propose that less bulk Na^+^ is reabsorbed in these tubules in undernourished rats because the lower transport demand in lower‐sized animals. However, it is remarkable that whereas the (Na^+^+K^+^)ATPase of RBD rats decreased by 30% with respect to CTRL, the decrease in body mass was 60%. In other words, when body mass is considered, the specific activity of (Na^+^+K^+^)ATPase activity is much higher in RBD‐fed rats. Conversely, upregulation of the one order of magnitude lower ouabain‐resistant ATPase means that the fine‐tuned Na^+^ reabsorption across the proximal epithelium mediated by this enzyme is increased, possibly contributing to the progressive genesis of arterial hypertension (Bełtowski et al., 2007).

Concerning rostafuroxin, the influence of the drug on proximal tubules (Na^+^+K^+^)ATPase (Figure 7a) matches the Na^+^ balance in CTRL rats, and the pronounced rostafuroxin‐induced inhibition encountered in the undernourished group could contribute to the normalization of systolic blood pressure. On the other hand, the positive Na^+^ balance in the RBD rostafuroxin‐treated rats needs to point to Figure 4c, which shows a very low Na^+^ excretion that cannot be explained by the effects of the drug on proximal tubules (Na^+^+K^+^)ATPase, but rather by an influence on the pump resident other segments, such as the distal tubule, which plays a central role in the genesis of hypertension and the regulation of bodily Na^+^ handling (Gonsalez et al., 2023).

Figure 8 shows that the drug completely normalized the arterial pressure in the RBD group without influence in CTRL rats and these facts could be discussed on the basis of three ideas: (i) the structural and functional undernutrition‐induced abnormalities proposed to occur in renal tissue on the basis of the Lim et al. (2000) hypothesis above discussed—which alter the responses of the two Na^+^‐transporting ATPases to rostafuroxin (Figure 7a,b)—are not present in arteries such as thoracic aorta and mesenteric arteries; (ii) rostafuroxin seems to act on these vessels only when a “prohypertensive tissular microenvironment” has developed (Dias et al., 2014), such as described in hypertensive rats (Dias et al., 2014; Ferrandi et al., 2002; Wenceslau & Rossoni, 2014; and this study); (iii) removal of silent Na^+^ promoted by rostafuroxin from the water‐free stores represented by glycosaminoglycans from the endothelial surface and from the perivascular interstitium (Olde Engberink et al., 2015; Reynertson et al., 1986) could also underpin the antihypertensive effect of rostafuroxin in undernourished rats. Even though the K^+^ content of RBD is lower than the CTRL diet, the undernourished rats did not present hypokalemia, and rostafuroxin did not modify [K^+^]_pls_; evidence that RBD‐fed rats control the dietary deficiency of this mineral that seems to not play any role in the genesis of their hypertension.

It is also noticeable that in the case of RBD rats, there is a total dissociation between Na^+^ balance and (Na^+^+K^+^)ATPase in both untreated and untreated animals (compare Figure 5a and Figure 7a). The results with undernourished rats are compatible with the idea that migration of the (Na^+^+K^+^)ATPase from the membrane and abnormal anchoring to cytoskeletal proteins has occurred, thus downregulating this pump (Simonini et al., 2018). The opposite is true when we look at the ouabain‐resistant Na^+^‐ATPase (Figure 7b), which its influence on the Na^+^ balance—despite its importance in body Na^+^ handling—is not evident due to its lower activity (compare ordinates in Figure 7a,b).

Finally, one mechanism deserves discussion. In one type of spontaneously hypertensive rats with high levels of endogenous ouabain, rostafuroxin did not interact with the RAAS (Ferrandi et al., 2002) and, therefore, the antihypertensive actions of the drug seem not to antagonize RAAS to prevent the genesis of hypertension. In the case of RBD undernourished rats, the cortical renal tubulointerstitium has more than four times the number of angiotensin II‐positive cells found in CTRL rats (Silva, Muzi‐Filho, et al., 2014), evidence of an increased tissular RAAS activity. Moreover, rostafuroxin‐sensitive hypertension they develop (Figure 8) is also completely normalized by the administration of losartan (Pereira‐Acácio et al., 2022), an antagonist of type 1 angiotensin II receptors (Timmermans et al., 1995), suggesting an interaction between rostafuroxin and RAAS. In terms of actions in vessels (such as the thoracic aorta and mesenteric arteries), rostafuroxin appears to have opposite effects and the same final targets as RAAS. It has been proposed that endogenous ouabain is regulated by type 2 angiotensin II receptors (Dmitrieva & Doris, 2002; Laredo et al., 1997; Schoner & Scheiner‐Bobis, 2007) which initiate the branch of RAAS that antagonizes the type 1 angiotensin II pathway (Miura et al., 2010). Thus, the cross‐talk between rostafuroxin‐sensitive signaling pathways and RAAS deserves further studies. Moreover, the possibility of cortical lesions that aggravate hypertension in chronic needs to be considered because low‐protein intake decreases the glomerular filtration rate and leads to more significant tubular injury and inflammation (Fotheringham et al., 2021).

## 5 CONCLUSION

We demonstrated that rostafuroxin exerts antihypertensive effects in undernourished hypertensive rats without influence in normonourished normotensive rats. The present study also provides evidence demonstrating that undernutrition provoked by a low‐protein, low‐salt, hypercaloric diet modifies the response of caloric and albumin metabolism, Na^+^ distribution, Na^+^ and water balance, and the activity of renal Na^+^‐transporting ATPases to the drug. There is also evidence regarding the involvement of endogenous cardiotonic steroids in the genesis of rostafuroxin‐sensitive hypertension in undernourished rats. For these reasons, the comprehension of the molecular mechanisms of the selective antihypertensive effect of the drug in these animals could open new avenues for the treatment of high arterial blood pressure in normonourished and undernourished humans.

## AUTHOR CONTRIBUTIONS

Amaury Pereira‐Acácio, João P. M. Veloso‐Santos, Humberto Muzi‐Filho, and Adalberto Vieyra conceived and designed research. Amaury Pereira‐Acácio, João P. M. Veloso‐Santos, Danilo Alves‐Bezerra, and Glória Costa‐Sarmento performed experiments. Amaury Pereira‐Acácio, João P. M. Veloso‐Santos, Humberto Muzi‐Filho, and Adalberto Vieyra analyzed data. Amaury Pereira‐Acácio, João P. M. Veloso‐Santos, Danilo Alves‐Bezerra, Glória Costa‐Sarmento, Humberto Muzi‐Filho, and Adalberto Vieyra interpreted the results of the experiments. Amaury Pereira‐Acácio, João P. M. Veloso‐Santos, and Adalberto Vieyra prepared figures. Amaury Pereira‐Acácio, João P. M. Veloso‐Santos, Humberto Muzi‐Filho, and Adalberto Vieyra drafted manuscript. Amaury Pereira‐Acácio, João P. M. Veloso‐Santos, Humberto Muzi‐Filho, and Adalberto Vieyra edited and revised the manuscript. Amaury Pereira‐Acácio, João P. M. Veloso‐Santos, Danilo Alves‐Bezerra, Glória Costa‐Sarmento, Humberto Muzi‐Filho, and Adalberto Vieyra approved the final version of the manuscript.

## FUNDING INFORMATION

This work was supported by Brazilian National Council for Scientific and Technological Development/CNPq (grant nos. 470266/2014 and 440544/2018‐1 to A.V.), Carlos Chagas Filho Foundation for Research Support of the State of Rio de Janeiro/FAPERJ (grant no. E‐26/210.890/2019 to A.V.), Coordination of Superior Level Staff Improvement/CAPES (grant nos. 88887.124150/2014‐00 and 88887.320213/2019‐00 to H.M‐F. and 88887.374390/2019‐00 and 88887.634142/2021‐00 to A.P‐A.).

## CONFLICT OF INTEREST STATEMENT

The authors declare no conflict of interest. The funders had no role in the design of the study; in the collection, analyses, or interpretation of data; in the writing of the manuscript; or in the decision to publish the results.

## Data Availability

The data presented in this study are available on request from the corresponding author.
